# Supplementary material for: Epitaxial Growth and Structural Characterizations of MnBi2Te4 Thin Films in Nanoscale
Source: Nanomaterials (Basel). 2021 Dec 7;11(12):3322. doi: 10.3390/nano11123322 (PMC8703544; doi:10.3390/nano11123322)
Supplement: Supplementary file 1 [file nanomaterials-11-03322-s001.zip › nanomaterials-1464090-supplementary.pdf]

Supplementary Materials

Epitaxial Growth and Structural Characterizations of $MnBi_2Te_4$ Thin Films in Nanoscale

Shu-Hsuan Su [1], Jen-Te Chang [1], Pei-Yu Chuang [1], Ming-Chieh Tsai [1], Yu-Wei Peng [1], Min Kai Lee [1], Cheng-Maw Cheng [2,3,4,5,*] and Jung-Chung Andrew Huang [1,5,*]

1 Department of Physics, National Cheng Kung University, Tainan 701, Taiwan;
macg0510@yahoo.com.tw (S.H.S.); c44051079@gs.ncku.edu.tw (J.-T.C.); chuang.py@nsrrc.org.tw (P.-Y.C.); maggie089621@gmaIl.com (M.-C.T.); g73582@gmail.com (Y.-W.P.);
anion3143@hotmail.com (M.K.L.)
2 National Synchrotron Radiation Research Center, Hsinchu 300, Taiwan
3 Department of Physics, National Sun Yat-sen University, Kaohsiung 80424, Taiwan
4 Graduate Institute of Applied Science and Technology, National Taiwan University of Science and Technology, Taipei 106335, Taiwan
5 Taiwan Consortium of Emergent Crystalline Materials, Ministry of Science and Technology, Taipei 10601, Taiwan
* Correspondence: makalu@nsrrc.org.tw (C.-M.C.); jcahuang@mail.ncku.edu.tw (J.-C.A.H.)

Supplementary Note 1. TEM-EDX spectrum of $MnBi_2Te_4$ film.

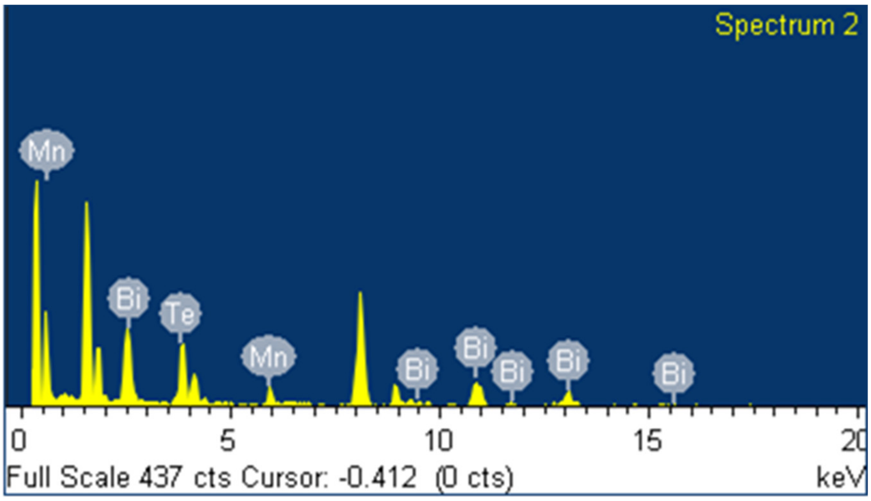

Figure S1. TEM-EDX spectrum recorded on the $MnBi_2Te_4$ film.

Typical TEM-EDX analysis of the atomic ratios of Bi, Mn and Te of the sample; the results are summarized in Table S1.

Table S1. Atomic ratio of Bi, Mn and Te of the sample.

**Element**	**Mn**	**Bi**	**Te**
**Atomic (at %)**	***13.97***	**30.29**	**55.74**
